# Comparing robot-assisted vs. laparoscopic proctectomy for rectal cancer surgical and oncological outcomes

**DOI:** 10.3389/fsurg.2025.1628649

**Published:** 2025-08-13

**Authors:** Wenpeng Wang, Jia Liu, Jiefu Wang, Jinghao Huang, Junfeng Wang

**Affiliations:** ^1^ Department of Colorectal Oncology, Tianjin Medical University Cancer Institute and Hospital, National Clinical Research Center for Cancer, Tianjin’s Clinical Research Center for Cancer, Tianjin Key Laboratory of Digestive Cancer, Tianjin, China; ^2^ Division of Hepatobiliary and Pancreas Surgery, Department of General Surgery, Shenzhen People's Hospital (The First Affiliated Hospital, Southern University of Science and Technology; The Second Clinical Medical College, Jinan University), Shenzhen, China

**Keywords:** laparoscopy, proctectomy, rectal neoplasms, robotic surgical procedures, survival analysis

## Abstract

**Background:**

Robotic-assisted proctectomy (RAP) is increasingly used for rectal cancer, but its long-term benefits over laparoscopic proctectomy (LP) remain debated. While RAP offers technical advantages, its clinical equivalence requires further validation, particularly in anatomically challenging cases.

**Methods:**

We conducted a retrospective analysis of all eligible patients who underwent RAP or LP for rectal cancer at Tianjin Medical University Cancer Institute and Hospital between 2019 and 2024.

**Results:**

In the overall cohort, RAP demonstrated significantly longer operative times (246.69 vs. 174.53 min, *p* < 0.001), greater blood loss (109.77 vs. 57.58 ml, *p* < 0.001), and higher costs (117,030.88 vs. 81,054.16 yuan, *p* < 0.001) compared to LP, with only a marginally shorter postoperative stay (8.47 vs. 8.64 days, *p* < 0.05). In terms of postoperative complications, RAP showed a trend towards fewer overall Clavien-Dindo Grade ≥ III complications (1.2% vs. 6.6%) compared to LP, although this difference was not statistically significant (*p* = 0.064). There were no significant differences in disease-free survival (DFS) (*p* = 0.575) or overall survival (OS) (*p* = 0.619) between the two groups. For the subgroup analysis of rectal cancers ≤ 5 cm from the anus, RAP achieved superior surgical precision, with 100% negative circumferential resection margin (CRM) (vs. 87.1% in LP, *p* = 0.042) and 100% complete mesorectal integrity (vs. 83.9% in LP, *p* = 0.053), alongside faster functional recovery (time to first flatus: 3.47 vs. 3.90 days, *p* = 0.034; time to urination: 2.10 vs. 2.65 days, *p* = 0.007). Recurrence rates were lower with RAP (10% vs. 19.4%), though survival outcomes remained similar between RAP and LP (*p* = 0.253)

**Conclusion:**

While RAP incurs longer operative times and higher costs, it demonstrates superior precision in anatomically complex cases, evidenced by improved CRM status and mesorectal preservation. Although survival outcomes remain comparable, RAP's advantages in functional recovery and potential recurrence reduction warrant further investigation.

## Introduction

Rectal cancer is one of the most prevalent malignancies worldwide, with surgery remaining the cornerstone of curative treatment. Before the introduction of total mesorectal excision (TME), local recurrence rates were alarmingly high, ranging from 30%–38% (1). The pioneering work of Dr. Heald in developing TME dramatically improved outcomes, reducing 5-year local recurrence to 3.7% and achieving 5-year disease-free survival (DFS) rates of approximately 80% (2).

While open TME represented a major advancement in rectal cancer surgery, subsequent technological refinements—including high-definition cameras and improved energy devices—have enhanced visualization and enabled minimally invasive approaches to achieve superior short-term outcomes like reduced pain, shorter hospitalization, and faster recovery compared to open techniques (3). Minimally invasive approaches (laparoscopic and robotic TME) have since emerged, offering enhanced visualization and precision in pelvic dissection. The use of vascular clips and advanced energy devices has further improved the safety of critical structure preservation (middle rectal artery, mesorectal fascia, and pelvic autonomic nerves) (4, 5).

The recent REAL trial—the first multicenter randomized controlled trial (RCT) with long-term oncological outcomes—has redefined the role of robotic-assisted proctectomy (RAP), demonstrating statistically superior 3-year locoregional recurrence and DFS compared to laparoscopic proctectomy (LP) for middle/low rectal cancer (6). This breakthrough complements earlier findings from the COLRAR trial, which showed comparable complete TME rates between RAP (80.7%) and LP (77.1%) approaches but superior circumferential resection margin (CRM) negativity with RAP (100% vs. 93.9%) in neoadjuvant-treated patients (7). The ROLARR trial, the seminal RCT comparing RAP and LP, demonstrated comparable conversion rates and pathological outcomes overall, though subgroup analyses suggested RAP's advantages in technically challenging cases (e.g., male pelvis, low tumors) (8).

Despite these advances, critical knowledge gaps persist. Existing research has predominantly focused on short-term surgical outcomes, and the cost-effectiveness implications of robotic surgery's demonstrated oncological superiority remain unquantified (9). This study aims to address these gaps by providing a comprehensive comparison of both short-term surgical outcomes and long-term survival rates between RAP and LP, while evaluating the value proposition of robotic technology in rectal cancer surgery.

## Patients and methods

### Study design

This retrospective study analyzed 177 patients with rectal cancer treated at Tianjin Medical University Cancer Institute and Hospital between November 2019 and June 2024. The surgical approach selection between RAP and LP was determined by three key factors: patient preference, family financial capacity, and surgeon's technical assessment. Primary clinical variables included operative parameters, postoperative morbidity, short-term oncological and long-term survival outcomes. Ethical approval was granted by the Institutional Review Board of Tianjin Medical University Cancer Institute and Hospital (Approval No. bc20240922).

### Patient inclusion and exclusion criteria

Patients eligible for this study met all the following criteria: (1) histologically confirmed rectal adenocarcinoma with surgical indications; (2) either non-metastatic (M0) disease or metastatic (M1) disease with liver metastases considered completely treatable with curative-intent resection or radiofrequency ablation following multidisciplinary team assessment (MDT); (3) primary tumors technically amenable to minimally invasive resection, including cases presenting with obstruction; and (4) expressed willingness to undergo either robotic or laparoscopic approach after detailed counseling regarding both techniques. The exclusion criteria included incomplete data, severe comorbidities or life-limiting chronic illnesses, poor treatment adherence, and lack of ethical standards.

### Standardized preoperative evaluation and surgical procedures

All patients underwent a comprehensive preoperative assessment to confirm surgical suitability, including: (1) endoscopic evaluation with colonoscopy and biopsy for histopathological confirmation, (2) imaging workup consisting of pelvic MRI for local tumor staging (assessing mesorectal fascia involvement and nodal status) and contrast-enhanced CT of the chest/abdomen to exclude distant metastases, (3) laboratory tests measuring gastrointestinal tumor markers (CEA, CA19-9, CA72-4, etc.) and routine blood parameters, and (4) MDT review to finalize therapeutic decisions. All cases met predefined surgical criteria.

Following this standardized evaluation, all patients underwent TME via either anterior resection [AR, including low (LAR) or high (HAR)] or abdominoperineal resection (APR). All anastomoses were performed using an end-to-end double-stapled technique with transanal insertion of circular staplers (24–28 mm, sized to bowel diameter and tumor level) and abdominal placement of the anvil.

### Total vs. conventional neoadjuvant and adjuvant therapy protocols

Total neoadjuvant therapy (TNT) was defined as the administration of both systemic chemotherapy and pelvic radiotherapy prior to surgery, without planned postoperative adjuvant chemotherapy. This approach, aligned with the National Comprehensive Cancer Network (NCCN) Clinical Practice Guidelines in Oncology for Rectal Cancer (https://www.nccn.org/guidelines/nccn-guidelines), aims to enhance tumor downstaging, improve the likelihood of achieving an R0 resection, and reduce the risk of distant metastasis. Patients were considered eligible for TNT if they had low-lying, locally advanced rectal cancer and/or exhibited a high risk of local recurrence or distant metastasis. High-risk features included one or more of the following factors: clinical T4 stage, extramural vascular invasion (EMVI) and/or tumor deposits identified on MRI, threatened involvement of the mesorectal fascia, or risk of invasion into the intersphincteric plane. The TNT protocol consisted of induction chemotherapy (4 cycles of XELOX regimen: oxaliplatin 130 mg/m^2^ on day 1 plus capecitabine 1,000 mg/m^2^ twice daily on days 1–14, every 3 weeks) followed by concurrent chemoradiotherapy (pelvic radiotherapy 45–50.4 Gy in 25–28 fractions with concurrent capecitabine 825 mg/m^2^ twice daily on radiation days). Surgery was uniformly performed 6–8 weeks after completing neoadjuvant therapy.

For conventional neoadjuvant therapy, eligible patients with clinical T3/T4 or node-positive disease who did not meet TNT criteria received either: (1) pelvic radiotherapy (45–50.4 Gy in 25–28 fractions) with concurrent capecitabine (825 mg/m^2^ twice daily on radiation days), or (2) chemotherapy-only with the XELOX regimen (oxaliplatin 130 mg/m^2^ on day 1 plus capecitabine 1,000 mg/m^2^ twice daily on days 1–14, every 3 weeks) for 4 cycles when radiotherapy was contraindicated. Surgical intervention followed 6–8 weeks post-treatment completion.

For adjuvant treatment, patients meeting the following criteria received 6–8 cycles of XELOX (same dosing as the neoadjuvant regimen), initiated within 4 weeks postoperatively: (1) those who underwent upfront surgery with pathological stage II disease exhibiting high-risk features [including poorly differentiated histology, lymphovascular invasion, perineural invasion (PNI), or <12 lymph nodes examined] or stage III disease, or positive CRM; or (2) those who received conventional neoadjuvant therapy with ypStage II-III disease or positive CRM.

### Pathological evaluation

TME quality was assessed by pathological examination, including: (1) distal resection margin (DRM): measured as the closest tumor-free distance from the distal edge of the specimen (≥1 cm recommended in rectal cancer) (10); (2) positive CRM: defined as tumor distance to the nearest non-peritonealized surgical margin ≤1 mm (11); (3) mesorectal integrity: graded as complete (intact mesorectum, Grade 3), near-complete (moderate defects, Grade 2), or incomplete (major defects, Grade 1) according to Nagtegaal criteria (12); (4) EMVI: defined as tumor cells within blood/lymphatic vessels beyond the muscularis propria (reported as present/absent); (5) PNI: identified as tumor infiltration along nerve sheaths (reported as present/absent).

### Follow-up protocol

#### Follow-up duration and schedule

Patients underwent regular surveillance including enhanced CT scans (chest/abdomen/pelvis), colonoscopy, and tumor marker monitoring (CEA, CA199, CA724). Follow-up intervals were structured as follows: quarterly for the first 2 postoperative years, semiannually during years 3–5, and annually thereafter.

#### Definition of survival outcomes

DFS: defined as the time from surgery to the first occurrence of any of the following events. Radiologically confirmed recurrence (enhanced CT/MRI or PET-CT showing a measurable lesion meeting RECIST 1.1 criteria). Distant metastasis (confirmed by either histopathology or dual imaging modalities). Overall survival (OS): defined as the time from surgery to death.

#### Censoring rules

Lost to follow-up: censored at the last documented follow-up date. Alive at study cutoff: censored on December 31, 2024. Non-cancer-related deaths: included in OS analysis but excluded from DFS events.

### Postoperative complications

Postoperative complications included anastomotic leakage, abdominal infection, urinary tract infection, bleeding, perineal infection, and intestinal obstruction and were assessed using the Clavien-Dindo classification (Grade I: no treatment beyond basic care; Grade II: complications needing pharmacological therapy; Grade III: surgical, endoscopic or other interventions; Grade IV: life-threatening complications demanding ICU management; Grade V: patient death) (13).

### Data collection

This study systematically collected the variables through electronic medical record review and prospective database entries. Operative variables were extracted from anesthesia records and surgical reports, pathological data from the institutional synoptic pathology reporting system, survival outcomes from the electronic medical records and family telephone follow-up, and complication data from standardized postoperative clinic documentation and re-admission records. Clinicopathological characteristics included age, sex, body mass index (BMI), AJCC pathological stage, histological differentiation, histopathologic types, and so on.

The primary endpoints included (1) surgical outcomes (operative time, blood loss, postoperative hospitalization), (2) short-term oncological results (DRM, CRM, mesorectal integrity, PNI, EMVI), and (3) long-term survival (DFS, OS). Secondary endpoints included postoperative complications (anastomotic leakage, surgical site infections, hemorrhage, and other events) and inpatient cost.

### Statistical analyses

Statistical analyses were performed using R 3.4.2 and SPSS (version 27.0; IBM Corp, USA). Categorical variables, including patient baseline characteristics, pathological outcomes, short-term oncological outcomes, postoperative complications, and clinicopathological factors, were expressed as numbers (percentages) and compared between RAP and LP groups using the chi-square test or Fisher's exact test for small sample sizes. Normally distributed continuous variables (e.g., operative time, blood loss) were presented as mean ± standard deviation (SD) and analyzed using *t*-test, while non-normally distributed data were evaluated with the Wilcoxon test but still reported as mean ± SD for consistency. Survival outcomes, including DFS and OS, were assessed through univariate Cox regression analysis to identify prognostic factors, with intergroup comparisons performed using Kaplan–Meier survival curves and log-rank tests. 1-, 3-, and 5-year cumulative survival rates were derived from Kaplan–Meier estimates. A two-sided *p*-value < 0.05 was considered statistically significant for all analyses.

## Results

### Patient characteristics

Table 1 provides an overview of the features of the 177 patients in the RAP and LP groups. There were 86 and 91 patients in the RAP and LP groups, respectively. The mean age was 59 year-old (31–71) in the RAP group and 61 (34–77) in the LP group, with a balanced sex distribution. There were no significant differences in age, sex, BMI between the two groups. There were also no significant differences observed between the groups in terms of histopathology (degree of histological differentiation, histopathologic type, tumor deposit, and AJCC TNM Stage). No significant differences were observed in neoadjuvant therapy and surgical approaches (including AR and APR surgery) (*p* > 0.05 for all). There was no significant difference in stoma formation between RAP and LP (*p* = 0.625). Temporary stoma rates were 43.0% (RAP) vs. 36.3% (LP), permanent stoma rates were 7.0% vs. 6.6%, and no stoma rates were 50.0% vs. 57.1%, respectively. Regarding the pathologic types, 152 cases were adenocarcinomas, and 25 cases were mixed adenocarcinomas including mucinous adenocarcinoma, sig-ring cell carcinoma, or other types. Four patients in the M1 stage with liver metastases were eligible for surgery or radiofrequency ablation. Sixteen patients who underwent neoadjuvant therapy in the two groups had mid-low rectal cancer at T3 stage or higher or with regional lymph node metastasis. Surgery was performed following neoadjuvant therapy with a therapeutic response of partial response or stable disease. No perioperative deaths occurred in either of the groups. Overall, there was no significant difference in the distribution of clinicopathological characteristics between RAP and LP groups (*p* > 0.05 for all; Table 1).

**Table 1:** Baseline clinicopathological characteristics of rectal cancer patients undergoing RAP versus LP.

Variable	RAP (*n* = 86)	LP (*n* = 91)	Chi-Square	*P*
Age (year-old)			1.521	0.217
<60	40 (46.5%)	34 (37.4%)		
≥60	46 (53.5%)	57 (62.6%)		
Sex			0.002	0.965
Female	40 (46.5%)	41 (45.1%)		
Male	46 (53.5%)	50 (54.9%)		
BMI classification (kg/m^2^)			1.373	0.503
<18.5	3 (3.5%)	2 (2.2%)		
18.5–24	62 (72.1%)	60 (65.9%)		
> 24	21 (24.4%)	29 (31.9%)		
Differentiation			4.720	0.094
Low	10 (11.6%)	22 (24.2%)		
Middle	72 (83.7%)	65 (71.4%)		
Unspecified	4 (4.7%)	4 (4.4%)		
Histology			2.480	0.115
Adenocarcinoma	78 (90.7%)	74 (81.3%)		
Mixed adenocarcinoma^a^	8 (9.3%)	17 (18.7%)		
AJCC pT stage			1.151	0.765
T1	8 (9.3%)	9 (9.9%)		
T2	25 (29.1%)	21 (23.1%)		
T3	45 (52.3%)	54 (59.3%)		
T4	8 (9.3%)	7 (7.7%)		
AJCC pN stage			1.719	0.423
N0	54 (62.8%)	63 (69.2%)		
N1	21 (24.4%)	15 (16.5%)		
N2	11 (12.8%)	13 (14.3%)		
AJCC pM stage			0.000	1.000
M0	84 (97.7%)	89 (97.8%)		
M1	2 (2.3%)	2 (2.2%)		
AJCC pTNM stage			1.628	0.653
I	26 (30.2%)	23 (25.3%)		
II	28 (32.6%)	38 (41.8%)		
III	30 (34.9%)	28 (30.8%)		
IV	2 (2.3%)	2 (2.2%)		
Tumor deposit			0.028	0.868
No	71 (82.6%)	77 (84.6%)		
Yes	15 (17.4%)	14 (15.4%)		
Neoadjuvant therapy			2.044	0.153
No	75 (87.2%)	86 (94.5%)		
Yes	11 (12.8%)	5 (5.5%)		
Surgery approach			0.000	1.000
AR	80 (93.0%)	85 (93.4%)		
APR	6 (7.0%)	6 (6.6%)		
Stoma formation			0.941	0.625
Temporary	37 (43.0%)	33 (36.3%)		
Permanent	6 (7.0%)	6 (6.6%)		
No	43 (50.0%)	52 (57.1%)		

RAP, robot-assisted proctectomy; LP, laparoscopic proctectomy; AJCC, American Joint Committee on Cancer; pT, pathological primary tumor extent; pN, pathological regional lymph node involvement; pM, pathological distant metastasis status; BMI, body mass index; AR, anterior resection; APR, abdominoperineal resection.

^a^ Mixed adenocarcinomas including mucinous adenocarcinoma, signet ring cell carcinoma and other pathological types.

### Perioperative surgical details and short-term oncological outcomes

The mean operative time was significantly longer for the RAP at 246.69 min compared to 174.53 min for the LP (*p* < 0.001). Blood loss was also notably higher in the RAP group (mean: 109.77 ml) than in the LP group (mean: 57.58 ml, *p* < 0.001). Despite the RAP group experiencing longer surgery time and greater blood loss, their postoperative hospital stay was significantly shorter than that of the LP group (mean: 8.47 vs. 8.64 days, *p* < 0.05). The RAP group also incurred higher hospitalization costs compared to the LP group (mean: 117,030.88 vs. 81,054.16 yuan, *p* < 0.001). However, the total number of lymph nodes retrieved was comparable between the groups (mean: 16.30 vs. 15.84, *p* = 0.257). Similarly, no significant difference was observed in the time to first flatus (mean: 3.59 days for RAP vs. 3.79 days for LP, *p* = 0.846; Table 2).

**Table 2:** Comparison of perioperative outcomes between RAP and LP.

Variable	RAP [mean (SD)]	LP [mean (SD)]	*P*
Operation time (min)	246.69 ± 71.41	174.53 ± 66.44	<0.001
Blood loss (ml)	109.77 ± 97.44	57.58 ± 60.91	<0.001
Total lymph nodes harvested	16.30 ± 6.23	15.84 ± 7.03	0.257
Time to passage of flatus (days)	3.59 ± 0.69	3.79 ± 0.66	0.846
Postoperative hospital stay (days)	8.47 ± 2.70	8.64 ± 3.79	0.049
Inpatient cost (yuan)	117,030.88 ± 16,578.68	81,054.16 ± 14,357.60	<0.001

SD, standard deviation; RAP, robot-assisted proctectomy; LP, laparoscopic proctectomy.

Table 3 showed that the RAP and LP approaches showed comparable short-term oncological outcomes, with no significant differences in DRM (*p* = 0.683), CRM positivity (*p* = 0.279), mesorectal integrity (*p* = 0.221), EMVI (*p* = 0.938), or PNI rates (*p* = 0.385). Although RAP demonstrated numerically lower CRM positivity (2.3% vs. 6.6%) and higher complete mesorectal excision rates (94.2% vs. 85.7% for Grade 3), these differences were not statistically significant (*p* > 0.05 for all).

**Table 3:** Comparison of short-term pathological outcomes between RAP and LP.

Variable	RAP (*n* = 86)	LP (*n* = 91)	Chi-Square	*P*
DRM (cm)			0.579	0.683
<1	2 (2.3%)	4 (4.4%)		
≥1	84 (97.7%)	87 (95.6%)		
CRM			1.866	0.279
Negative	84 (97.7%)	85 (93.4%)		
Positive	2 (2.3%)	6 (6.6%)		
Mesorectal integrity			3.477	0.221
Grade 1	2 (2.3%)	5 (5.5%)		
Grade 2	3 (3.5%)	8 (8.8%)		
Grade 3	81 (94.2%)	78 (85.7%)		
EMVI			0.006	0.938
Negative	75 (87.2%)	79 (86.8%)		
Positive	11 (12.8%)	12 (13.2%)		
PNI			0.755	0.385
Negative	79 (91.9%)	80 (87.9%)		
Positive	7 (8.1%)	11 (12.1%)		

DRM, distal resection margin; CRM, circumferential resection margin; EMVI, extramural vascular invasion; PNI, perineural invasion; Mesorectal integrity was graded as complete (Grade 3), near-complete (Grade 2), or incomplete (Grade 1) per Nagtegaal criteria.

### Postoperative complications

The postoperative complication rates were 10.5% (9/86) in the RAP group and 13.2% (12/91) in the LP group, with no meaningful statistical distinction between the two groups, as indicated by a *p*-value of 0.576. No considerable variations were identified between the two groups in the frequency of postoperative complications, including postoperative bleeding, abdominal infection, perineal infection, urinary system infection, intestinal obstruction, anastomotic leakage and anal incontinence (*p* > 0.05 for all; Table 4).

**Table 4:** Comparison of postoperative complications between RAP and LP.

Variable	RAP (*n* = 86)	LP (*n* = 91)	Chi-Square	*P*
Complications	9 (10.5%)	12 (13.2%)	0.313	0.576
Postoperative bleeding	2 (2.3%)	3 (3.3%)	0.000	1.000
Abdominal infection	2 (2.3%)	1 (1.1%)	0.000	0.961
Perineal infection	1 (1.2%)	0 (0%)	0.000	0.977
Urinary system infection	1 (1.2%)	2 (2.2%)	0.000	1.000
intestinal obstruction	0 (0%)	1 (1.1%)	0.000	1.000
Anastomotic leakage	3 (3.5%)	4 (4.4%)	0.000	1.000
Anal incontinence	0 (0%)	1 (1.1%)	0.000	1.000

RAP, robot-assisted proctectomy; LP, laparoscopic proctectomy.

Table 5 revealed differences in complication profiles between RAP and LP. Overall, RAP showed a non-significant trend toward fewer high-grade complications (Grade ≥ III: 1.2% vs. 6.6%, *p* = 0.064) compared to LP. Although the absolute rate of anastomotic leakage was higher in the LP group (Grade ≥ III: 3.3% vs. 1.2%), this difference did not reach statistical significance (*p* = 0.621).

**Table 5:** Incidence of postoperative complications by Clavien-Dindo classification between RAP and LP.

Variable	RAP (*n* = 86)		LP (*n* = 91)		*P* ^a^
	Grade ≤ II	Grade ≥ III	Grade ≤ II	Grade ≥ III	
Complications	8 (9.3%%)	1 (1.2%)	6 (6.6%)	6 (6.6%)	0.064
Postoperative bleeding	2 (2.3%)	0 (0%)	1 (1.1%)	2 (2.2%)	0.498
Abdominal infection	2 (2.3%)	0 (0%)	1 (1.1%)	0 (0%)	-
Perineal infection	1 (1.2%)	0 (0%)	0 (0%)	0 (0%)	-
Urinary infection	1 (1.2%)	0 (0%)	2 (2.2%)	0 (0%)	-
intestinal obstruction	0 (0%)	0 (0%)	0 (0%)	1 (1.1%)	1.000
Anastomotic leakage	2 (2.3%)	1 (1.2%)	1 (1.1%)	3 (3.3%)	0.621
Anal incontinence	0 (0%)	0 (0%)	1 (1.1%)	0 (0%)	-

RAP, robot-assisted proctectomy; LP, laparoscopic proctectomy; Clavien-Dindo classification: Grade ≤ II complications required observation or medications; Grade ≥ III involved surgical, endoscopic or other interventions (III), ICU care (IV), or death (V).

^a^ Comparison of Grade ≥ III complications between RAP and LP.

### Long-term comparison of outcomes

All enrolled patients were systematically followed up until December 2024. The median follow-up duration for all patients was 36 months (range: 6–62 months). As shown in Table 6, univariate analysis of DFS revealed no significant difference between RAP and LP (*p* = 0.587). Advanced AJCC pTNM stage, tumor deposits, positive CRM, EMVI, and neoadjuvant therapy were associated with worse DFS (*p* < 0.05 for all), while mesorectal integrity grade 3 correlated with improved outcomes (*p* < 0.001). Age, sex, DRM, and PNI showed no significant impact for DFS (*p* > 0.05 for all).

**Table 6:** Univariate analysis of DFS for patients with rectal cancer.

Variable	Group	HR (95% CI)	SE	Z-score	*P*
Surgical approach	LP	Ref.			
	RAP	0.82 (0.39–1.70)	0.365	−0.543	0.587
Age (year-old)	<60	Ref.			
	≥60	1.45 (0.72–2.92)	0.361	1.048	0.295
Sex	Female	Ref.			
	Male	0.52 (0.25–1.06)	0.369	−1.804	0.071
AJCC pTNM	I	Ref.			
	II	4.14 (0.93–18.47)	0.782	1.862	0.063
	III	10.10 (2.44–41.83)	0.744	3.19	0.001
	IV	48.10 (4.24–545.06)	1.287	3.127	0.002
Tumor deposit	No	Ref.			
	Yes	3.47 (1.69–7.15)	0.376	3.377	0.001
DRM	<1	Ref.			
	≥1	0.79 (0.09–6.97)	1.017	−0.215	0.830
CRM	Negative	Ref.			
	Positive	4.47 (1.53–13.09)	0.61	2.734	0.006
Mesorectal integrity	Grade 1	Ref.			
	Grade 2	0.47 (0.16–1.37)	0.919	−1.388	0.165
	Grade 3	0.20 (0.11–0.38)	0.741	−5.047	< 0.001
EMVI	Negative	Ref.			
	Positive	4.30 (1.85–1.00)	0.43	3.383	0.001
PNI	Negative	Ref.			
	Positive	2.77 (0.99–7.77)	0.536	1.938	0.053
Neoadjuvant therapy	No	Ref.			
	Yes	2.66 (1.10–6.42)	0.455	2.169	0.03

HR, hazard ratio; CI, confidence interval; SE, standard error; AJCC, American joint committee on cancer; pT, pathological primary tumor extent; pN, pathological regional lymph node involvement; pM, pathological distant metastasis status; DRM, distal resection margin; CRM, circumferential resection margin; EMVI, extramural vascular invasion; PNI, perineural invasion; DFS, disease-free survival; Mesorectal integrity was graded as complete (Grade 3), near-complete (Grade 2), or incomplete (Grade 1) per Nagtegaal criteria.

Univariate analysis of OS showed comparable outcomes between RAP and LP (*p* = 0.628). Advanced AJCC stages (III: *p* = 0.007; IV: *p* < 0.001), tumor deposits (*p* = 0.005), and EMVI (*p* = 0.001) significantly reduced OS, while margin status (CRM: *p* = 0.163; DRM: *p* = 0.501) and other variables (mesorectal integrity: *p* = 0.147; PNI: *p* = 0.124; neoadjuvant therapy: *p* = 0.052) showed no significant impact for OS (Table 7).

**Table 7:** Univariate analysis of OS for patients with rectal cancer.

Variable	Group	HR (95% CI)	SE	Z-score	*P*
Surgical approach	LP	Ref.			
	RAP	0.80 (0.33–1.96)	0.447	−0.484	0.628
Age (year-old)	<60	Ref.			
	≥60	1.08 (0.47–2.50)	0.437	0.184	0.854
Sex	Female	Ref.			
	Male	0.60 (0.25–1.43)	0.451	−1.151	0.25
AJCC pTNM	I	Ref.			
	II	5.59 (0.67–46.5)	1.097	1.593	0.111
	III	16.16 (2.18–119.99)	1.037	2.72	0.007
	IV	1,080.36 (75.15–15,532.30)	1.726	5.136	< 0.001
Tumor deposit	No	Ref.			
	Yes	3.46 (1.46–8.20)	0.467	2.827	0.005
DRM	<1	Ref.			
	≥1	0.45 (0.04–4.58)	1.028	−0.673	0.501
CRM	Negative	Ref.			
	Positive	2.97 (0.64–13.66)	0.747	1.396	0.163
Mesorectal integrity	Grade 1	Ref.			
	Grade 2	0.82 (0.12–5.71)	1.168	−0.199	0.842
	Grade 3	0.26 (0.04–1.61)	1.033	−1.449	0.147
EMVI	Negative	Ref.			
	Positive	5.06 (1.88–13.60)	0.523	3.21	0.001
PNI	Negative	Ref.			
	Positive	2.53 (0.78–8.24)	0.625	1.54	0.124
Neoadjuvant therapy	No	Ref.			
	Yes	2.59 (0.99–6.74)	0.514	1.947	0.052

HR, hazard ratio; CI, confidence interval; SE, standard error; AJCC, American joint committee on cancer; pT, pathological primary tumor extent; pN, pathological regional lymph node involvement; pM, pathological distant metastasis status; DRM, distal resection margin; CRM, circumferential resection margin; EMVI, extramural vascular invasion; PNI, perineural invasion; OS, overall survival; Mesorectal integrity was graded as complete (Grade 3), near-complete (Grade 2), or incomplete (Grade 1) per Nagtegaal criteria.

**Figure 1:**
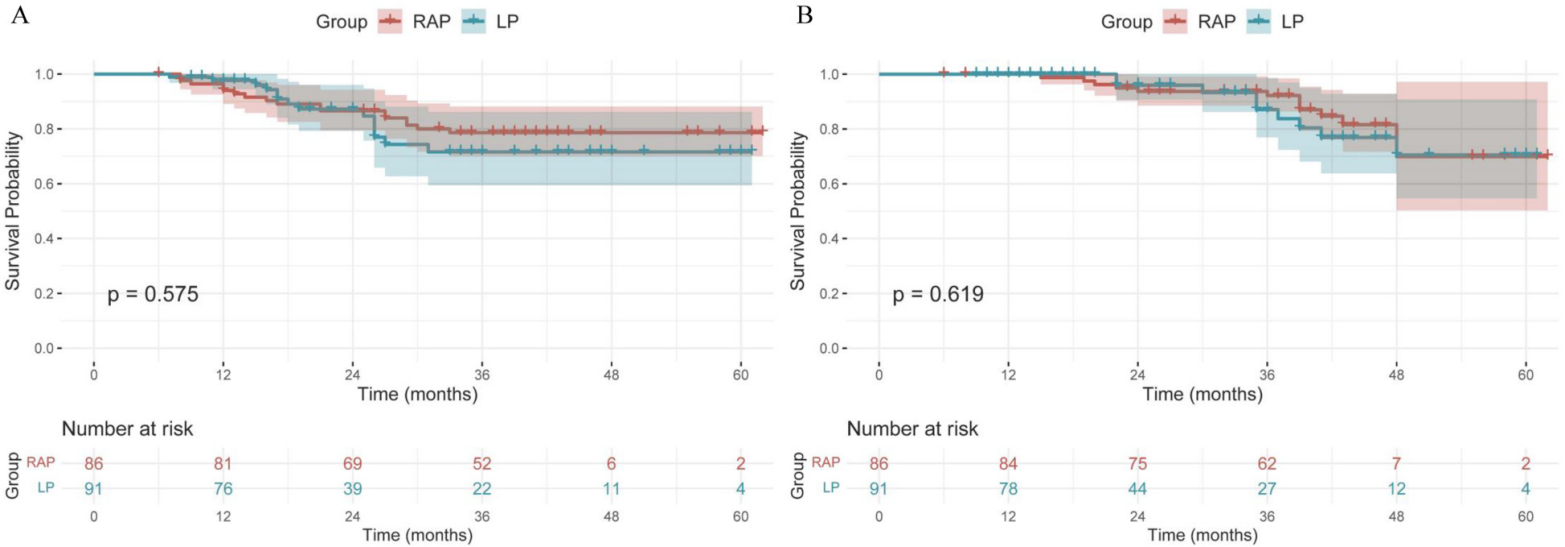
Kaplan–Meier curves of DFS **(A)** and OS **(B)** for RAP and LP group. RAP, robot-assisted proctectomy; LP, laparoscopic proctectomy; DFS, disease-free survival; OS, overall survival.

Using the log-rank test for comparison, the cumulative survival rates showed no statistically significant differences between RAP and LP groups for either DFS (*p* = 0.575) or OS (*p* = 0.619) (Figure 1). The 5-year DFS rates were 78.5% for RAP vs. 71.2% for LP, while 5-year OS rates were 69.5% vs. 68.7%, respectively. Both surgical approaches demonstrated comparable long-term survival outcomes, with all inter-group comparisons being non-significant by log-rank analysis. Notably, while RAP showed numerically higher 1-year and 3-year survival rates for OS (98.8% vs. 95.7% at 1-year; 92.2% vs. 83.4% at 3-year), these differences did not reach statistical significance (Table 8).

**Table 8:** Cumulative survival rates of DFS and OS at 1, 3, and 5 years by log-rank method.

Survival	Group	1-year	3-year	5-year	*P*
DFS	RAP	92.8%	78.5%	78.5%	0.575
	LP	97.7%	71.2%	71.2%	
OS	RAP	98.8%	92.2%	69.5%	0.619
	LP	95.7%	83.4%	68.7%	

DFS, disease-free survival; OS, overall survival; RAP, robot-assisted proctectomy; LP, laparoscopic proctectomy.

### Clinical and pathological factors in rectal cancer ≤5 cm: a comparison between RAP and LP groups

The comparison between the RAP and LP groups for rectal cancer ≤5 cm from the anus revealed some notable differences in clinicopathological factors (Table 9). While the distribution of sex and BMI showed no significant differences between the groups (*p* > 0.05 for both), the CRM status and mesorectal integrity were notably distinct. The RAP group had no positive CRM, whereas 12.9% of the LP group did (*p* = 0.042), and RAP group patients exhibited Grade 3 mesorectal integrity compared to 83.9% in the LP group (*p* = 0.053). These findings suggest better surgical outcomes in terms of resection margins and mesorectal preservation for the RAP group.

**Table 9:** Comparison of clinicopathological factors in RAP and LP groups for rectal cancer ≤5 cm from the anus.

Variable	RAP (*n* = 86)	LP (*n* = 91)	*P*
Sex			0.246
Female	12 (40.0%)	17 (54.8%)	
Male	18 (60.0%)	14 (45.2%)	
BMI classification (kg/m^2^)			0.363
<18.5	1 (3.3%)	0 (0.0%)	
18.5–24	24 (80.0%)	22 (71.0%)	
>24	5 (16.7%)	9 (29.0%)	
Surgery approach			0.949
AR	24 (80.0%)	25 (80.6%)	
APR	6 (20.0%)	6 (19.4%)	
DRM (cm)			0.352
<1	2 (6.7%)	4 (12.9%)	
≥1	28 (93.3%)	27 (87.1%)	
CRM			0.042
Negative	30 (100%)	27 (87.1%)	
Positive	0 (0.0%)	4 (12.9%)	
Mesorectal integrity			0.053
Grade 1	0 (0.0%)	3 (9.7%)	
Grade 2	0 (0.0%)	2 (6.5%)	
Grade 3	30 (100%)	26 (83.9%)	
Recurrence			0.253
Yes	3 (10.0%)	6 (19.4%)	
No	27 (90.0%)	25 (80.6%)	
Operation time (min)	227.67 ± 63.89	201.61 ± 76.69	0.115
Blood loss (ml)	82.00 ± 44.52	68.39 ± 40.17	0.215
Postoperative hospital stay (days)	7.97 ± 1.83	8.94 ± 4.74	0.300
Time to passage of flatus (days)	3.47 ± 0.78	3.90 ± 0.79	0.034
Time to spontaneous urination (days)	2.10 ± 0.61	2.65 ± 0.88	0.007

RAP, robot-assisted proctectomy; LP, laparoscopic proctectomy; AJCC, American Joint Committee on Cancer; pT, pathological primary tumor extent; DRM, distal resection margin; CRM, circumferential resection margin; Mesorectal integrity was graded as complete (Grade 3), near-complete (Grade 2), or incomplete (Grade 1) per Nagtegaal criteria.

Regarding postoperative recovery, the RAP group demonstrated significantly quicker recovery in terms of time to passage of flatus (3.47 ± 0.78 vs. 3.90 ± 0.79 days, *p* = 0.034) and time to spontaneous urination (2.10 ± 0.61 vs. 2.65 ± 0.88 days, *p* = 0.007). Intriguingly, there were no significant differences in operation time, blood loss, or postoperative hospital stay (*p* > 0.05 for all). The recurrence rates were also assessed, with 10% of the RAP group experiencing recurrence compared to 19.4% in the LP group, though this difference was not statistically significant (*p* = 0.253). These results suggest that RAP offers potential advantages in recovery time compared to LP.

## Discussion

Robotic surgery is an innovative approach in minimally invasive rectal cancer resection. Ergonomic advantages, AI-based assistants, operational flexibility and precision, and more realistic visual systems have promoted the humanization and intelligent development of minimally invasive surgery (14, 15). However, there are also influencing factors such as long operation time and high hospitalization costs. Previous studies have estimated learning curves ranging from 18–60 procedures for mastering robotic surgery (16). The robotic surgical system is more complicated than a conventional laparoscopic system. The docking times in operations using the da Vinci robotic surgical system ranged from 7–22 min. While these preparatory steps provided more operational flexibility and precision for the operation and reduced postoperative complications, they also prolonged the overall operation time (17). Similarly, in this study, the operative time for RAP was longer than that for LP. In a study by Baek et al., involving 182 patients who underwent robotic rectal cancer surgery, factors such as high BMI, preoperative chemoradiotherapy, and lower tumor level were identified as contributors to prolonged surgical duration (18). Interestingly, the study found no significant correlation between pelvimetric parameters and operation time, suggesting that pelvic anatomical measurements may not play a substantial role in influencing the length of robotic procedures.

Most studies have shown that RAP using the da Vinci Surgical System is associated with reduced blood loss (17). However, in this study, there was significantly more blood loss in the RAP group than that in the LP group in the overall cohort. Robotic surgery typically has a longer operative time, which may increase the risk of bleeding. A longer operation time meant longer exposure and tissue manipulation time, potentially leading to more blood vessel damage and bleeding. In conventional laparoscopic surgery, intraoperative bleeding can be controlled using conventional methods such as electrocoagulation and clipping. Although robotic surgery has similar functions, owing to the complexity of the operation or unforeseen technical problems during the operation, bleeding management may not be as effective as laparoscopic surgery. This technical difference may be one reason for the higher bleeding rate in the robotic surgery group. Among our 86 RAP cases, 31 (36%) utilized the da Vinci Si system, which has recognized limitations in image resolution (standard HD vs. Xi's 3D-HD) and instrument articulation compared to the Xi platform. This technological disparity may have impacted hemostatic precision in these earlier cases. This finding aligns with previous studies reporting greater blood loss in robotic colorectal surgery compared to laparoscopic approaches (Robotic surgery: 147.8 vs. Laparoscopic surgery: 103.9 ml) (19). Intriguingly, our subgroup analysis suggests that RAP did not show a significant difference in terms of operative time and blood loss when compared to LP for low rectal cancer. In addition, the increase in blood loss did not reach the level of clinically requiring blood transfusion or negatively affect the patient's postoperative recovery, indicating that RAP is still acceptable in terms of surgical safety. However, future studies can further explore how to reduce operative time and intraoperative blood loss by optimizing the surgical procedures and techniques.

The length of postoperative hospital stay is often closely related to the degree of surgical trauma, postoperative complications, postoperative pain, postoperative rehabilitation process, and the hospital management system (20). RAP can be used to perform more delicate operations in complex anatomical areas and reduce damage to the surrounding tissues and nerves. In theory, robotic surgery should reduce surgical trauma and shorten postoperative recovery time. In our study, there was a significant but small difference in postoperative hospital stay between RAP and LP (mean 8.47 days vs. 8.64 days), which may be related to individual differences, postoperative management and recovery processes in the hospital. Even minor complications (postoperative infection, anastomotic leakage, urinary dysfunction, etc.) may prolong hospital stay. Although postoperative pain was not evaluated in this study, multiple studies have shown that robotic surgery has certain advantages in reducing postoperative pain and preserving urinary function and defecation, which can reduce hospital stay (21). Our subgroup analysis in this study suggests that for rectal cancer ≤5 cm from the anus, the time to first passage of flatus and time to spontaneous urination were both significantly better in the RAP compared to the LP. This suggests that RAP may have an advantage in preserving pelvic nerves after low rectal cancer surgery.

The quality of lymphadenectomy and negative margins are key indicators for evaluating the quality of rectal cancer surgery (22). Studies have shown that high-quality lymphadenectomy improves the accuracy of tumor staging, thereby affecting the survival rate (23). In our study, no significant difference was observed in the lymph nodes yield between two groups. Furthermore, no significant difference was observed in the incidence of postoperative complications between the two groups. However, there was a trend toward fewer Clavien-Dindo Grade ≥ III complications in the RAP group compared with the LP group (1.2% vs. 6.6%), although larger sample sizes are needed to confirm whether this difference reaches statistical significance.

Our study found comparable oncological outcomes for DRM, CRM or mesorectal integrity between RAP and LP in the overall cohort. However, our subgroup analysis of low rectal cancers (≤5 cm from the anal verge) revealed advantages for CRM negativity and complete mesorectal excision in RAP group compared to LP group. Although recurrence rates did not reach statistical significance, the numerical trend suggests potential oncologic benefits in anatomically challenging cases, likely attributable to robotic precision in confined pelvic spaces. These findings are consistent with previously published studies demonstrating the advantages of robotic surgery for low rectal cancer resection (7, 8).

The factors affecting survival rate are very complex and involve multiple aspects such as tumor histology, tumor stage, surgical approaches, and so on. In our study, there was no significant difference in the distribution of tumor stages and differentiation between RAP and LP. Although recent RCTs have demonstrated that RAP reduces recurrence and prolongs DFS compared to LP while maintaining comparable OS, our univariate Cox analysis did not reveal significant differences in either DFS or OS between the two groups, possibly due to the limited sample size (6).

The cost is the main reason why RAP has not been widely promoted. Several studies have shown that the cost of RAP is 1.3–2.5 times higher than that of LP. However, if precise surgical indications are applied, RAP may be more cost-effective in the long term. An analysis of claims data of 16,541 cases reported that RAP resulted in lower overall costs within one-year post-surgery in the health care system in Japan (24).

A key limitation of this study is the small sample size, particularly in low-rectal cancer, limiting the evaluation of robotic surgery's advantages in confined spaces. Additionally, crucial data on postoperative sexual and bladder function are missing. The inferior hypogastric nerve and pelvic plexus are vital for preserving these functions, requiring careful handling of the rectal sidewall. Lastly, the retrospective design may introduce data bias, highlighting the need for prospective studies to confirm these findings.

In addition, the comparable learning curve to achieve proficiency in RAP may have influenced the outcomes of this study, as operation time and blood loss are typically higher during the initial learning phase. In our study, both participating surgeons completed formal robotic training at Prince of Wales Hospital, Hong Kong, in 2017. Following certification, they performed 15 robotic rectal cancer surgeries within 90 days post-training. By study initiation, both had surpassed the initial learning curve, with ≥30 prior robotic cases, ensuring technical proficiency. A review of 34 studies indicated that 39 cases were needed for a surgeon to reach the expert level in RAP (25). More accessible and cost-effective surgical robots could enhance robotic procedures and oncological outcomes, and the integration of automation and AI in RAP is expected to further improve surgical accuracy and efficiency.

## Conclusion

This study indicates that RAP did not show substantial advantages in perioperative outcomes, postoperative complications, or short- and long-term oncological results compared to LP in the overall cohort. However, after performing subgroup analysis specifically on low rectal cancers, RAP demonstrated notable benefits, particularly in achieving negative CRM and preserving mesorectal integrity. Furthermore, RAP facilitated faster postoperative recovery, including quicker time to first flatus and spontaneous urination. These findings emphasize the precision and flexibility of RAP in managing low rectal cancer in confined pelvic spaces.

## Data Availability

The raw data supporting the conclusions of this article will be made available by the authors, without undue reservation.

## References

[1] Cho MS Bae HW Kim NK. Essential knowledge and technical tips for total mesorectal excision and related procedures for rectal cancer. Ann Coloproctol. (2024) 40:384–411. 10.3393/ac.2024.00388.0055 39228201 PMC11375228

[2] Heald RJ Husband EM Ryall RD. The mesorectum in rectal cancer surgery–the clue to pelvic recurrence? Br J Surg. (1982) 69:613–6. 10.1002/bjs.1800691019 6751457

[3] Chew MH Yeh YT Lim E Seow-Choen F. Pelvic autonomic nerve preservation in radical rectal cancer surgery: changes in the past 3 decades. Gastroenterol Rep (Oxf). (2016) 4:173–85. 10.1093/gastro/gow023 27478196 PMC4976685

[4] Jiang W-Z Xu J-M Xing J-D Qiu H-Z Wang Z-Q Kang L Short-term outcomes of laparoscopy-assisted vs open surgery for patients with low rectal cancer: the LASRE randomized clinical trial. JAMA Oncol. (2022) 8:1607–15. 10.1001/jamaoncol.2022.4079 36107416 PMC9478880

[5] Quintana JM Anton-Ladislao A Lázaro S Gonzalez N Bare M de Larrea NF Outcomes of open versus laparoscopic surgery in patients with rectal cancer. Int J Colorectal Dis. (2018) 33:99–103. 10.1007/s00384-017-2925-2 29110087

[6] Feng Q Yuan W Li T Tang B Jia B Zhou Y Robotic vs. laparoscopic surgery for middle and low rectal cancer: the REAL randomized clinical trial. JAMA. (2025) 334(2):136–48. 10.1001/jama.2025.8123 40455621 PMC12131176

[7] Park JS Lee SM Choi G-S Park SY Kim HJ Song SH Comparison of laparoscopic versus robot-assisted surgery for rectal cancers: the COLRAR randomized controlled trial. Ann Surg. (2023) 278:31–8. 10.1097/SLA.0000000000005788 36594748

[8] Jayne D Pigazzi A Marshall H Croft J Corrigan N Copeland J Effect of robotic-assisted vs, conventional laparoscopic surgery on risk of conversion to open laparotomy among patients undergoing resection for rectal cancer: the ROLARR randomized clinical trial. JAMA. (2017) 318:1569–80. 10.1001/jama.2017.7219 29067426 PMC5818805

[9] Yu HY Friedlander DF Patel S Hu JC. The current status of robotic oncologic surgery. CA Cancer J Clin. (2013) 63:45–56. 10.3322/caac.21160 23161385

[10] Song SH Park JS Choi G-S Seo AN Park SY Kim HJ Impact of the distal resection margin on local recurrence after neoadjuvant chemoradiation and rectal excision for locally advanced rectal cancer. Sci Rep. (2021) 11(1):22943. 10.1038/s41598-021-02438-1 34824330 PMC8617265

[11] Liu Q Luo D Cai S Li Q Li X. Circumferential resection margin as a prognostic factor after rectal cancer surgery: a large population-based retrospective study. Cancer Med. (2018) 7(8):3673–81. 10.1002/cam4.1662 29992773 PMC6089167

[12] Nagtegaal ID van de Velde CJH van der Worp E Kapiteijn E Quirke P van Krieken JHJM. Macroscopic evaluation of rectal cancer resection specimen: clinical significance of the pathologist in quality control. J Clin Oncol. (2002) 20(7):1729–34. 10.1200/JCO.2002.07.010 11919228

[13] Clavien PA Barkun J de Oliveira ML Vauthey JN Dindo D Schulick RD The Clavien-Dindo classification of surgical complications: five-year experience. Ann Surg. (2009) 250(2):187–96. 10.1097/SLA.0b013e3181b13ca2 19638912

[14] Wee IJY Kuo LJ Ngu JC. A systematic review of the true benefit of robotic surgery: ergonomics. Int J Med Robot. (2020) 16:e2113. 10.1002/rcs.2113 32304167

[15] Eoh KJ Kim TJ Park JY Kim HS Paek J Kim YT. Robot-assisted versus conventional laparoscopic surgery for endometrial cancer: long-term comparison of outcomes. Front Oncol. (2023) 13:121937. 10.3389/fonc.2023.1219371 PMC10540847 37781200

[16] Dal Moro F Secco S Valotto C Artibani W Zattoni F. Specific learning curve for port placement and docking of da Vinci((R)) surgical system: one surgeon’s experience in robotic-assisted radical prostatectomy. J Robot Surg. (2012) 6:323–7. 10.1007/s11701-011-0315-2 27628472

[17] Yang Y Wang F Zhang P Shi C Zou Y Qin H Robot-assisted versus conventional laparoscopic surgery for colorectal disease, focusing on rectal cancer: a meta-analysis. Ann Surg Oncol. (2012) 19:3727–36. 10.1245/s10434-012-2429-9 22752371

[18] Baek SJ Kim CH Cho MS Bae SU Hur H Min BS Robotic surgery for rectal cancer can overcome difficulties associated with pelvic anatomy. Surg Endosc. (2015) 29(6):1419–24. 10.1007/s00464-014-3818-x 25159651

[19] Hu D-p Zhu X-l Wang H Liu W-h Lv Y-c Shi X-l Robotic-assisted versus conventional laparoscopic surgery for colorectal cancer: short-term outcomes at a single center. Indian J Cancer. (2021) 58:225–31. 10.4103/ijc.IJC_86_19 33753624

[20] Fiore JF Jr Bialocerkowski A Browning L Faragher IG Denehy L. Criteria to determine readiness for hospital discharge following colorectal surgery: an international consensus using the Delphi technique. Dis Colon Rectum. (2012) 55:416–23. 10.1097/DCR.0b013e318244a8f2 22426265

[21] Khajeh E Aminizadeh E Dooghaie Moghadam A Nikbakhsh R Goncalves G Carvalho C Outcomes of robot-assisted surgery in rectal cancer compared with open and laparoscopic surgery. Cancers (Basel). (2023) 15:839. 10.3390/cancers15030839 36765797 PMC9913667

[22] Bae JH Song J Yoo RN Kim JH Kye B-H Lee IK Robotic lateral pelvic lymph node dissection could harvest more lateral pelvic lymph nodes over laparoscopic approach for mid-to-low rectal cancer: a multi-institutional retrospective cohort study. Biomedicines. (2023) 11:1556. 10.3390/biomedicines11061556 37371651 PMC10295381

[23] Baik SM Lee RA. Weighing the benefits of lymphadenectomy in early-stage colorectal cancer. Ann Surg Treat Res. (2023) 105:245–51. 10.4174/astr.2023.105.5.245 38023437 PMC10648610

[24] Yoshihara H Igarashi A D'Attilio D Shin M Mizutani K. EE163 analysis of healthcare resource use of the robotic surgery system for rectal cancer in Japan. Value Health. (2022) 25:S84–5. 10.1016/j.jval.2022.09.413

[25] Jiménez-Rodríguez RM Rubio-Dorado-Manzanares M Díaz-Pavón JM Reyes-Díaz ML Vazquez-Monchul JM Garcia-Cabrera AM Learning curve in robotic rectal cancer surgery: current state of affairs. Int J Colorectal Dis. (2016) 31:1807–15. 10.1007/s00384-016-2660-0 27714517

